# Molecular identification of *Fusarium* species in commercial vanilla and crop wild relatives in Colombia

**DOI:** 10.1111/1758-2229.70038

**Published:** 2024-11-11

**Authors:** Jayerlin Rodríguez‐Bastidas, Santiago Manrique‐Barros, Donald Riascos‐Ortiz, Ana T. Mosquera‐Espinosa, Nicola S. Flanagan

**Affiliations:** ^1^ Department of Natural Sciences and Mathematics Pontificia Universidad Javeriana Cali Cali Colombia; ^2^ Research Center Obonuco Corporación Colombiana de Investigación Agropecuaria‐AGROSAVIA Nariño Colombia

## Abstract

Vanilla is an economically important crop for low‐lying humid tropical regions, but cultivated plants face serious phytosanitary problems. Fusarium wilt is a devastating disease affecting vanilla crops, caused by the fungal pathogens *Fusarium oxysporum* f. sp. *vanillae* (Fov) and *F. oxysporum* f. sp. *radicis‐vanillae* (Forv), part of the *F. oxysporum* species complex (FOSC). We characterized 29 fungal isolates from a vanilla crop and crop wild relatives (CWR) using molecular (EF1‐α and ITS‐rRNA loci) and morphological traits. *Fusarium* was the predominant genus, followed by *Colletotrichum* and *Clonostachys*. Four *Fusarium* species were identified: *F. oxysporum* (37.9%), *Fusarium solani* (20.7%), *Fusarium pseudocircinatum* (13.8%) and *Fusarium concentricum* (10.3%). The latter three species were isolated only from CWR and may represent latent pathogens. Fov was isolated from both the crop and CWR, while a Forv‐affiliated isolate was also found in a vanilla crop, marking the first report in the neotropical region. The EF1‐α locus provided greater genotype resolution, as well as having reference sequences for Forv. However, the fungal barcode ITS locus is widely applied. We recommend the continued use of both loci for *Fusarium* diagnosis in vanilla to facilitate early detection and the development of effective integrated crop management strategies.

## Graphical abstract

Fungal strains with affinity to both *Fusarium oxysporum* f. sp. *vanillae* (Fov) and *F. oxysporum* f. sp. *radicis‐vanillae* (Forv) were isolated from symptomatic and asymptomatic tissues of both commercial vanilla crops and crop wild relatives (CWR) in Colombia.

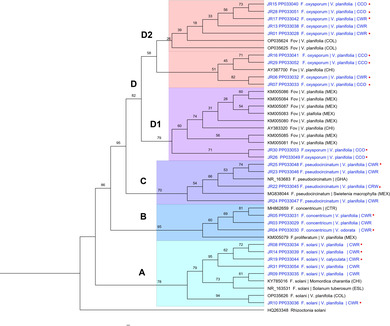

## INTRODUCTION

Vanilla is an aromatic compound of high commercial value due to its multiple applications in the food and cosmetic industries. The natural vanilla aroma is extracted from the cured fruits of species in the genus *Vanilla* subgen. *Xanata* sec. *Xanata* (Orchidaceae) native to the neotropical region (Soto Arenas & Cribb, 2010). The most widely cultivated species is *Vanilla planifolia* (Bory et al., 2008); however, other species within this section also have aromatic potential (de Oliveira et al., 2022; Pérez‐Silva et al., 2021).

Colombia is a centre of diversity for *Vanilla*, with 26 species reported, of which 21 belong to section *Xanata* (Flanagan et al., 2022; Flanagan & Mosquera‐Espinosa, 2016). These species, including native populations of *V. planifolia*, represent the vanilla crop wild relatives (CRW), an important agrobiodiversity resource for crop breeding initiatives. As some species also represent promising sources of natural vanilla, incipient cultivation initiatives are under way in circa *situm* conditions (Flanagan et al., 2019).

Planting material for the vanilla crop is mainly generated through vegetative propagation, which results in low levels of genetic diversity and promotes phytosanitary problems in commercial production systems, especially the attack of pathogenic microorganisms (Azofeifa‐Bolaños et al., 2014; Ramírez‐Mosqueda et al., 2019; Sharma & Marques, 2018). The diseases known as Fusarium wilt and stem and root rot are the main limiting factor of vanilla cultivation in producing countries such as Mexico (Adame‐García et al., 2015; Hernández‐Hernández, 2019), China (Xiong et al., 2017), Malaysia (Ujat et al., 2021), Reunion Islands and Madagascar (Koyyappurath et al., 2015). This pathology is predominantly associated with *Fusarium oxysporum*, although others species such as *Fusarium fujikuroi*, *Fusarium graminearum*, *Fusarium mangiferae*, *Fusarium napiforme*, *Fusarium solani*, *Fusarium proliferatum* and *Fusarium pseudocircinatum* are also reported as latent pathogens in the crop or fulfilling other ecological roles as endophytes or saprophytes (Adame‐García et al., 2015; Koyyappurath et al., 2015, 2016; Mosquera‐Espinosa et al., 2022; Pinaria et al., 2010, 2015).

*F. oxysporum*, which is part of the *F. oxysporum* species complex (FOSC), stands out as one of the major pathogens of global economic importance, with the ability to infect a wide variety of plant species (Lombard, Sandoval‐Denis, Cai, et al., 2019; Thangavel et al., 2021). FOSC is a polyphyletic evolutionary group that includes about 21 cryptic phylogenetic species, of which 15 have been officially described (Lombard, Sandoval‐Denis, Caiet al., 2019). The identification, description and nomenclature of FOSC strains are considered highly complex due to multiple and confusing subspecific classification systems, as well as the lack of type material for previously described species (D. R. Gordon & Martyn, 1997; Lombard, Sandoval‐Denis, Caiet al., 2019; O'Donnell, Kistler, et al., 1998).

Phytopathogenic strains of *F. oxysporum* are characterized by their ability to infect specific plant hosts (Lombard, Sandoval‐Denis, Lamprecht, et al., 2019). These isolates are known as special forms, an informal subspecies rank term that is not subject to the International Code of Nomenclature for algae, fungi and plants (ICN) (Lombard, Sandoval‐Denis, Lamprecht, et al., 2019; Thangavel et al., 2021), thus do not require the deposition of reference sequences in recognized databases such as GenBank (Turland et al., 2018). The scarcity of reference sequences complicates molecular diagnosis and phylogenetic inference, representing a major obstacle to providing reliable identities to special forms originating from diverse hosts in cultivation systems (Lombard, Sandoval‐Denis, Cai, et al., 2019; O'Donnell et al., 2004).

In vanilla, two special forms of *F. oxysporum* are reported as potentially devastating for this crop, inducing different symptomatologies. *F. oxysporum* f. sp. *vanillae* (Fov) (Adame‐García et al., 2015) spreads systemically through the vascular bundles, generating chlorosis and subsequent rotting from the root to the aerial organs of the plant (Koyyappurath et al., 2015; Mosquera‐Espinosa et al., 2022). On the contrary, *F. oxysporum* f. sp. *radicis‐vanillae* (Forv) is limited to root and root collar tissues, and results in eventual plant collapse due to water and nutrient deficiency (Koyyappurath et al., 2016).

To date, Forv has been reported by Koyyappurath et al. (2015, 2016) in Reunion Island, Madagascar, Indonesia and French Polynesia, whereas Fov has been reported more frequently in these countries and in most other vanilla‐producing countries (Adame‐García et al., 2015; Bory et al., 2008; Mosquera‐Espinosa et al., 2022; Pinaria et al., 2015). In the Neotropics, information about the diversity of species and special forms of *Fusarium* associated with vanilla is minimal (Manrique‐Barros et al., 2023; Mosquera‐Espinosa et al., 2022). Moreover, there are no studies that address the presence of Forv in this region.

Historically, species delimitation in the genus *Fusarium* is challenging due to the lack of a sexual stage and the instability of morphological characters commonly employed for species identification. Morphological characterization of *Fusarium* has focused exclusively on its asexual phase (O'Donnell, Kistler, et al., 1998, 2004; Thangavel et al., 2021). However, characteristics such as colony colour, type of mycelium, shape and size of conidia, as well as chlamydospore formation, are traits that may vary according to the culture medium and environmental conditions (Nelson, 1991; Thangavel et al., 2021). Therefore, it is necessary to look for more reliable alternatives that complement the morphological information and allow for making the right decisions for the integrated management of phytopathogenic species.

In the last decade, molecular characterization has allowed a better understanding of the genetic diversity within FOSC (Adame‐García et al., 2015; Azofeifa‐Bolaños et al., 2014; Flores‐de la Rosa et al., 2023; Ujat et al., 2021). The ITS‐rRNA region, encompassing the internal transcribed spacers ITS‐1 & ITS‐2, is considered the universal barcode marker for fungal identification down to the species level because it has conserved and variable regions (Crous et al., 2015; Schoch et al., 2012; Zhang et al., 2022) and has been used previously to characterize *F. oxysporum* f. sp. *vanillae* (Adame‐García et al., 2015; Mosquera‐Espinosa et al., 2022), and recently in metabarcoding studies on the vanilla microbiome (Carbajal‐Valenzuela et al., 2022). However, no data are available for the ITS‐rRNA locus of the special form *F. oxysporum* f. sp. *radicis‐vanillae* (Forv), which was genotyped using two loci—the translation elongation factor 1‐α (EF1‐α) and the ribosomal intergenic spacer (IGS) region (Koyyappurath et al., 2015). The EF1‐α locus possesses abundant polymorphic characters and can resolve intraspecific phylogenetic relationships within FOSC (Lombard, Sandoval‐Denis, Lamprecht, et al., 2019).

To facilitate the identification of isolates and relate the findings to other studies on FOSC in vanilla, in the present study, we aimed to characterize at the ITS‐rRNA and EF1‐α loci the *Fusarium* species and the special forms of *F. oxysporum* associated with symptomatic plant material from a vanilla crop and its wild relatives. We also aimed to provide baseline reference data for the future molecular identification of Fov and Forv fungal strains that may be present in emerging vanilla crops.

Consequently, the following hypotheses were proposed in this research: (1) In small‐scale vanilla agroforestry systems in Colombia, the expectation is for the coexistence of different *Fusarium* species, as well as the presence of special forms of *F. oxysporum*, Fov and Forv (Koyyappurath et al., 2015, 2016). (2) The combination of the ITS‐rRNA and EF1‐α sequence data will allow a precise identification of the special forms of *F. oxysporum* associated with vanilla, with differentiation between Fov and Forv. This approach is supported, based on previous research done in vanilla (Flores‐de la Rosa et al., 2023; Koyyappurath et al., 2015, 2016; Pinaria et al., 2015), as well as studies conducted in other crops such as *Carya illinoinensis* in Brazil (Poletto et al., 2020), *Stevia rebaudiana* in Mexico (Leyva‐Mir et al., 2018) and *Poncirus trifoliata* in China (Ma et al., 2023), where high accuracy has been demonstrated for the identification of *Fusarium* species by using the ITS and EF1‐α locus together.

## EXPERIMENTAL PROCEDURES

### Sampling and fungal isolates

Leaf and root tissue was collected from plants of *Vanilla* species under circa *situm* and agroforestry conditions in the state of Valle del Cauca, Colombia. Samples were taken from *V. planifolia* of commercial crop origin (CCO) and from crop wild relatives (CWR). CCO plants are derived from commercial crop but are found in a small agroforestry system in the municipality of Alcalá (Colombia), rather than in a monoculture system as *V. planifolia* is typically grown. Meanwhile, CWR are located in the municipalities of Buenaventura and Atuncela‐Dagua.

Three root samples presenting symptoms of dry rot or wilting of stems and roots were collected from *V. planifolia* from CCO. The remaining samples were taken from small necrotic lesions or asymptomatic leaf tissue from both CCO plants and CWR. The tissue fragments (~5 mm^2^) were extracted and superficially disinfested by immersing them in 70% ethanol for 1 min, 2.5% sodium hypochlorite for 30 s, 70% ethanol for 1 min and then washed with sterile distilled water (Mosquera‐Espinosa et al., 2010). Tissue sections were placed in Petri dishes containing Potato‐Dextrose Agar (PDA) culture medium at 50% of the commercial concentration of the product (Manrique‐Barros et al., 2023) and kept in incubation in complete darkness at 26 ± 1°C (Mosquera‐Espinosa et al., 2010, 2022).

### Morphological characterization of fungal isolates

The description of the macroscopic characteristics (colour and texture of the mycelium) of fungal isolates growing on 50% PDA was performed after a 10‐day incubation (Mosquera‐Espinosa et al., 2022). Microscopic characteristics (appearance and organization of conidia) were observed and photographed using a light microscope (Leica ICC50 W) at 40× and 100×. The size of microscopic characteristics (e.g., conidia) of the fungi was measured from 50 observation points, using the IMAGE J v.1.5.3.8 program (Mirghasempour et al., 2022; Zhang et al., 2022).

### DNA extraction

For DNA extraction, inoculum from each isolate was maintained in a 20 mL suspension of Potato‐Dextrose Broth Dextrose medium (PDB‐Difco brand, USA) for 10 days. Subsequently, 0.5 mL of mycelial growth was extracted, deposited and macerated in 1.5 mL Eppendorf tubes, 400 μL of SDS extraction buffer (200 mM Tris–HCl [pH 8], EDTA 10 mM [ph 8], 500 mM NaCl and 1% SDS) was added (Koyyappurath et al., 2016) and DNA purified following the method of Mahuku (2004). DNA integrity was determined by 1% agarose gel electrophoresis supplemented with EZ vision (5 μL) and SB 1X loading buffer; a UVP transilluminator (BioDoc‐ItTM Imaging System) was used for gel visualization.

### DNA amplification by PCR

DNA sequences were obtained from the partial regions of the ITS‐rRNA and EF1‐α genes, using the primer pairs ITS‐4/ITS‐5 (5′‐TCCTCCGCTTATTGATATATGC‐3′/5′‐GGAAGTAAAAGTCGTAACAAGG‐3′) (White et al., 1990) and EF1/EF2 (5′‐ATGGGTAAGGA (A/G) GACAAGAC‐3′/5′‐GGA(G/A) GTACCAGT (G/C) ATCATGTT‐3′) (O'Donnell, Kistler, et al., 1998), respectively. PCR reactions were prepared in a final volume of 50 μL by adding 5 μL of DNA + 45 μL of Master Mix (5 μL of Taq Buffer [KCl‐MgCl] 1× + 5 μL of MgCl_2_ 2, 5 mM + 5 μL DNTPs 0.2 mM + 5 μL Forward 0.2 mM primer + 5 μL Reverse 0.2 mM primer + 19.5 μL water + 0.5 μL Taq polymerase) (Koyyappurath et al., 2016). Amplification was performed on a T100 Thermal Cycler (Bio‐Rad). For ITS‐rRNA, the PCR conditions consisted of an initial denaturation at 94°C for 2 min, followed by 40 cycles at 94°C for 45 s, 55°C for 1 min, 72°C for 1 min and a final extension at 72°C for 5 min (Adame‐García et al., 2015). For the EF1‐α locus, PCR conditions consisted of an initial denaturation at 96°C for 5 min, followed by 35 cycles of denaturation at 96°C for 45 s, a banding phase at a gradient temperature between 50 and 57°C for 1 min, an extension at 72°C for 1 min and a final extension at 72°C for 7 min (Koyyappurath et al., 2016; Pinaria et al., 2015). The amplicons were run on a 1.5% agarose gel and purified by isopropanol precipitation. Sanger sequencing was performed for both directions at Macrogen Company (South Korea). Nucleotide sequences obtained in this study were deposited in GenBank under the accession numbers PP033028‐PP033054 for ITS‐rRNA, and PP754860‐PP754864, PP778402‐PP778410 y PP835900‐PP835912 for EF1‐α.

### Molecular data analysis

DNA sequences were aligned and edited to obtain a consensus sequence, and their quality was verified using Geneious software (version 6.1.8) through electropherogram analysis. Molecular identification was performed by BLASTn searches in the NCBI nucleotide database (http://blast.ncbi.nlm.nih.gov/), filtering accessions corresponding to type material. A similarity to accessions from a threshold of 97% or above was considered determinant for species identity (Vu et al., 2019). Sequence alignment for each locus was run using MAFFT software (v7.511) (Katoh et al., 2019).

Sequences from ITS‐rRNA and EF1‐α regions of vanilla‐associated *Fusarium* isolates reported by Adame‐García et al. (2015), Pinaria et al. (2015), Koyyappurath et al. (2016), Carbajal‐Valenzuela et al. (2022) and Mosquera‐Espinosa et al. (2022) were included in the alignment, with other accessions of interest extracted from GenBank (Table 1). A sequence from *Rhizoctonia solani* (HQ263348) was assigned as outgroup for ITS‐rRNA, while a sequence from *Colletotrichum horii* (GQ329693) was used for EF1‐α analysis. Phylogenetic analysis was performed with IQ‐TREE software (v. 1.6.12) that allows detection of the substitution model that best fits the data (Lam‐Tung et al., 2015; Minh et al., 2020). For the tree reconstruction, the maximum likelihood (MV) method was used based on the model chosen according to Bayesian Information Criterion (BIC) (Mosquera‐Espinosa et al., 2022). The bootstrap consensus tree was estimated from 10,000 replicates (Koyyappurath et al., 2016). Figtree (version 1.4.4) was used for tree visualization and editing (Robinson et al., 2016).

**TABLE 1:** GenBank accessions used as reference for the construction of phylogenetic trees.

Accession GenBank	Locus	Species	Host	Country	Reference
KM005079	ITS‐rRNA	*F. proliferatum*	*Vanilla planifolia*	Mexico	Adame‐García et al. (2015)
KM005080	ITS‐rRNA	*Fusarium oxysporum* f. sp. *vanillae*	*V. planifolia*	Mexico	Adame‐García et al. (2015)
KM005087	ITS‐rRNA	*F. oxysporum* f. sp. *vanillae*	*V. planifolia*	Mexico	Adame‐García et al. (2015)
KM005083	ITS‐rRNA	*F. oxysporum* f. sp. *vanillae*	*V. planifolia*	Mexico	Adame‐García et al. (2015)
KM005084	ITS‐rRNA	*F. oxysporum* f. sp. *vanillae*	*V. planifolia*	Mexico	Adame‐García et al. (2015)
KM005086	ITS‐rRNA	*F. oxysporum* f. sp. *vanillae*	*V. planifolia*	Mexico	Adame‐García et al. (2015)
KM005085	ITS‐rRNA	*F. oxysporum* f. sp. *vanillae*	*V. planifolia*	Mexico	Adame‐García et al. (2015)
KM005081	ITS‐rRNA	*F. oxysporum* f. sp. *vanillae*	*V. planifolia*	Mexico	Adame‐García et al. (2015)
OP035624	ITS‐rRNA	*F. oxysporum* f. sp. *vanillae*	*V. planifolia* (CWR)	Colombia	Mosquera‐Espinosa et al. (2022)
OP035625	ITS‐rRNA	*F. oxysporum* f. sp. *vanillae*	*V. planifolia*	Colombia	Mosquera‐Espinosa et al. (2022)
OP035626	ITS‐rRNA	*Fusarium solani*	*V. planifolia*	Colombia	Mosquera‐Espinosa et al. (2022)
MZ270651	ITS‐rRNA	*Clonostachys* sp.	*V. planifolia*	Mexico	Carbajal‐Valenzuela et al. (2022)
NR_163683	ITS‐rRNA	*Fusarium pseudocircinatum*	‐	Ghana	Vu et al. (2019)
MH862659	ITS‐rRNA	*Fusarium concentricum*	‐	Costa Rica	Vu et al. (2019)
NR_165993	ITS‐rRNA	*Clonostachys rosea* f. *catenulata*	‐	USA	Vu et al. (2019)
NR_163540	ITS‐rRNA	*Clonostachys solani* f. *nigrovirens*	‐	Netherlands	Vu et al. (2019)
AY383320	ITS‐rRNA	*F. oxysporum* f. sp. *vanillae*	*V. planifolia*	China	Wang et al. (2003a)
AY387700	ITS‐rRNA	*F. oxysporum* f. sp. *vanillae*	*V. planifolia*	China	Wang et al. (2003b)
NR_163531	ITS‐rRNA	*F. solani*	*Solanum tuberosum*	Slovenia	Schroers et al. (2016)
KY785016	ITS‐rRNA	*F. solani*	*Momordica charantia*	China	Wen and Guo (2018)
MG838044	ITS‐rRNA	*F. pseudocircinatum*	*Swietenia macrophylla*	Mexico	Santillán‐Mendoza et al. (2018)
KU378600	EF1‐α	*F. oxysporum* f. sp. *vanillae*	*V. planifolia*	Mexico	Flores‐de la Rosa et al. (2023)
KU378602	EF1‐α	*F. oxysporum* f. sp. *vanillae*	*V. planifolia*	Mexico	Flores‐de la Rosa et al. (2023)
KU378604	EF1‐α	*F. oxysporum* f. sp. *vanillae*	*V. planifolia*	Mexico	Flores‐de la Rosa et al. (2023)
KU378601	EF1‐α	*F. oxysporum* f. sp. *vanillae*	*V. planifolia*	Mexico	Flores‐de la Rosa et al. (2023)
KM065859	EF1‐α	*F. oxysporum* f. sp. *radicis‐vanillae*	*V. planifolia*	Reunion Island	Koyyappurath et al. (2016)
KM065857	EF1‐α	*F. oxysporum*	*V. planifolia*	Reunion Island	Koyyappurath et al. (2016)
KM065867	EF1‐α	*F. oxysporum* f. sp. *radicis‐vanillae*	*V. planifolia*	Reunion Island	Koyyappurath et al. (2016)
KM065851	EF1‐α	*F. oxysporum* f. sp. *radicis‐vanillae*	*V. planifolia*	Reunion Island	Koyyappurath et al. (2016)
KM065853	EF1‐α	*F. oxysporum* f. sp. *radicis‐vanillae*	*V. planifolia*	Reunion Island	Koyyappurath et al. (2016)
KM065846	EF1‐α	*F. oxysporum* f. sp. *radicis‐vanillae*	*V. planifolia*	Reunion Island	Koyyappurath et al. (2016)
KM065874	EF1‐α	*F. solani*	*V. planifolia*	Reunion Island	Koyyappurath et al. (2016)
KM115168	EF1‐α	*F. oxysporum* f. sp. *vanillae*	*V. planifolia*	Indonesia	Pinaria et al. (2015)
KM115169	EF1‐α	*F. oxysporum* f. sp. *vanillae*	*V. planifolia*	Indonesia	Pinaria et al. (2015)
KM115170	EF1‐α	*F. oxysporum* f. sp. *vanillae*	*V. planifolia*	Indonesia	Pinaria et al. (2015)
KM115177	EF1‐α	*F. oxysporum* f. sp. *vanillae*	*V. planifolia*	Reunion Island	Pinaria et al. (2015)
KM115178	EF1‐α	*F. oxysporum* f. sp. *vanillae*	*V. planifolia*	Reunion Island	Pinaria et al. (2015)
KM115179	EF1‐α	*F. oxysporum* f. sp. *vanillae*	*V. planifolia*	Mexico	Pinaria et al. (2015)
KM115180	EF1‐α	*F. oxysporum* f. sp. *vanillae*	*V. planifolia*	Mexico	Pinaria et al. (2015)
KM115181	EF1‐α	*F. oxysporum* f. sp. *vanillae*	*V. planifolia*	Indonesia	Pinaria et al. (2015)
KM115182	EF1‐α	*F. oxysporum* f. sp. *vanillae*	*V. planifolia*	Indonesia	Pinaria et al. (2015)
KM115183	EF1‐α	*F. oxysporum* f. sp. *vanillae*	*V. planifolia*	Indonesia	Pinaria et al. (2015)
KM115184	EF1‐α	*F. oxysporum* f. sp. *vanillae*	*V. planifolia*	Indonesia	Pinaria et al. (2015)
KM115185	EF1‐α	*F. oxysporum* f. sp. *vanillae*	*V. planifolia*	Mexico	Pinaria et al. (2015)
KM115186	EF1‐α	*F. oxysporum* f. sp. *vanillae*	*V. planifolia*	Reunion Island	Pinaria et al. (2015)
GQ425230	EF1‐α	*F. pseudocircinatum*	*V. planifolia*	Indonesia	Pinaria et al. (2015)
AF008484	EF1‐α	*F. oxysporum* f. sp. *batatas*	*Ipomoea batatas*	USA	O'Donnell et al. (2004)
AF008505	EF1‐α	*F. oxysporum* f. sp. *passiflorae*	*Passiflora edulis*	USA	O'Donnell, Cigelnik, et al. (1998)
AF008485	EF1‐α	*F. oxysporum* f. sp. *canariensis*	*Phoenix canariensis*	Spain	O'Donnell, Kistler, et al. (1998)
AF008487	EF1‐α	*F. oxysporum* f. sp. *cubense*	*Musa acuminata*	Australia	O'Donnell, Kistler, et al. (1998)
KU378599	EF1‐α	*F. oxysporum* f. sp. *vanillae*	*V. planifolia*	Mexico	Flores‐de la Rosa et al. (2023)
KU378595	EF1‐α	*F. oxysporum* f. sp. *vanillae*	*V. planifolia*	Mexico	Flores‐de la Rosa et al. (2023)
KU378589	EF1‐α	*F. oxysporum* f. sp. *vanillae*	*V. planifolia*	Mexico	Flores‐de la Rosa et al. (2023)
KU378596	EF1‐α	*F. oxysporum* f. sp. *vanillae*	*V. planifolia*	Mexico	Flores‐de la Rosa et al. (2023)
KU378590	EF1‐α	*F. oxysporum* f. sp. *vanillae*	*V. planifolia*	Mexico	Flores‐de la Rosa et al. (2023)
KU378591	EF1‐α	*F. oxysporum* f. sp. *vanillae*	*V. planifolia*	Mexico	Flores‐de la Rosa et al. (2023)
KU378597	EF1‐α	*F. oxysporum* f. sp. *vanillae*	*V. planifolia*	Mexico	Flores‐de la Rosa et al. (2023)
KU378598	EF1‐α	*F. oxysporum* f. sp. *vanillae*	*V. planifolia*	Mexico	Flores‐de la Rosa et al. (2023)
KT313611	EF1‐α	*F. solani*	*S. tuberosum*	Slovenia	Schroers et al. (2016)
AF160282	EF1‐α	*F. concentricum*	*Musa sapientum*	Costa Rica	O'Donnell, Cigelnik, et al. (1998)
AF160271	EF1‐α	*F. pseudocircinatum*	*Solanum* sp.	Ghana	Lima et al. (2009)
HM057295	EF1‐α	*F. oxysporum* f. sp. *lycopersici*	*Solanum lycopersicum*	USA	Huang (2009)
HM057328	EF1‐α	*F. oxysporum* f. sp. *radicis‐lycopersici*	*Solanum lycopersicum*	USA	Huang (2009)
HM057330	EF1‐α	*F. oxysporum* f. sp. *lycopersici*	*Solanum lycopersicum*	USA	Huang (2009)
HM057327	EF1‐α	*F. oxysporum* f. sp. *radicis‐lycopersici*	*Solanum lycopersicum*	USA	Huang (2009)
MN078955	EF1‐α	*F. oxysporum* f. sp. *narcissi*	*Narcissus* sp.	UK	Taylor et al. (2019)
KP964904	EF1‐α	*F. oxysporum* f. sp. *cepae*	*Allium cepa*	UK	Taylor et al. (2019)
KY775756	EF1‐α	*F. oxysporum* f. sp. *momordicae*	*M. charantia*	China	Guo et al. (2019)
KY775751	EF1‐α	*F. oxysporum* f. sp. *momordicae*	*M. charantia*	China	Guo et al. (2019)
MT305193	EF1‐α	*F. oxysporum* f. sp. *asparagi*	*Asparagus officinalis*	Spain	Brizuela et al. (2020)
MT568936	EF1‐α	*F. oxysporum* f. sp. *asparagi*	*A. officinalis*	Spain	Brizuela et al. (2020)
MN386728	EF1‐α	*F. oxysporum*	*Sansevieria trifasciata*	Malaysia	Kee et al. (2020)
MN386744	EF1‐α	*F. pseudocircinatum*	*S. trifasciata*	Malaysia	Kee et al. (2020)
GU737398	EF1‐α	*F. pseudocircinatum*	*Mangifera indica*	USA	Otero‐Colina et al. (2010)
AF333934	EF1‐α	*F. concentricum*	Suelo de *Pinus densiflora*	Japan	Aoki et al. (2001)

## RESULTS

### *Symptomatology of* Vanilla *spp. in field*

**FIGURE 1:**
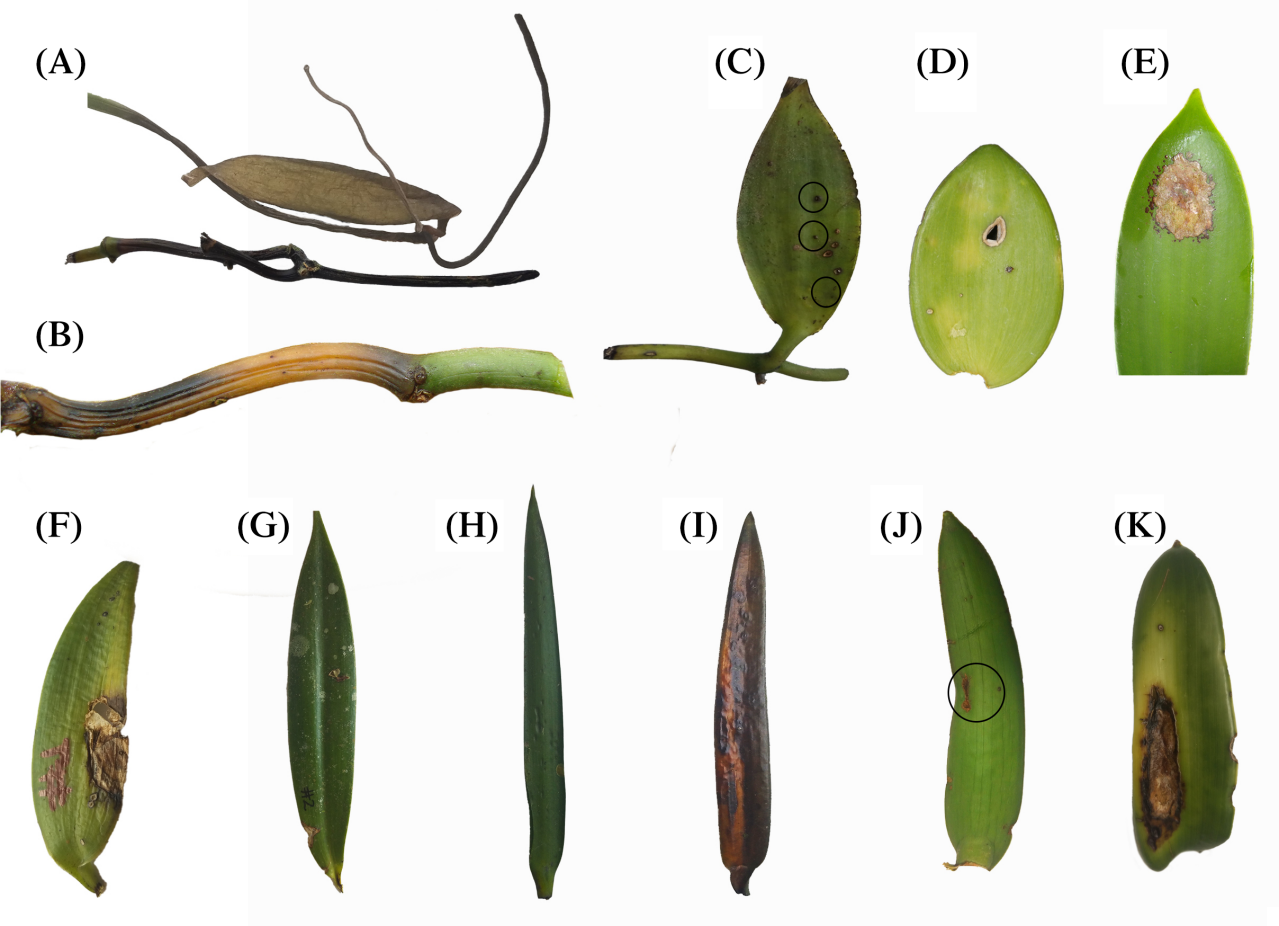
Symptoms in sampled tissue. (A) Dry rot of stem and roots. (B) Stem wilt and drying. (C) Black spots on leaf. (D) Chlorotic leaf spots. (E) Brown leaf spot. (F) Leaf wilt. (G, H) Asymptomatic leaf tissue. (I) Wet leaf rot. (J) Reddish‐brown lesion on leaf. (K) Wet‐appearing necrotic spot. (A–G) *Vanilla planifolia*. (H, I) *V* *anilla* *odorata*. (J, K) *V*. *calyculata*.

*V. planifolia* from CCO exhibited characteristic symptoms of Fusarium wilt, such as dry rot (Figure 1A) and wilt (Figure 1B,F) of stems and roots. In CWR plants under circa *situm* conditions, the disease severity was low, and symptom expression was higher on leaves compared with stems and roots. In addition, anthracnose symptoms such as the presence of black spots and brown spots with wet appearance and tissue softening were found on leaves of *V*. *planifoli*a and *Vanilla calyculata* (Figure 1E,K).

### Morphological characterization of fungal isolates

**FIGURE 2:**
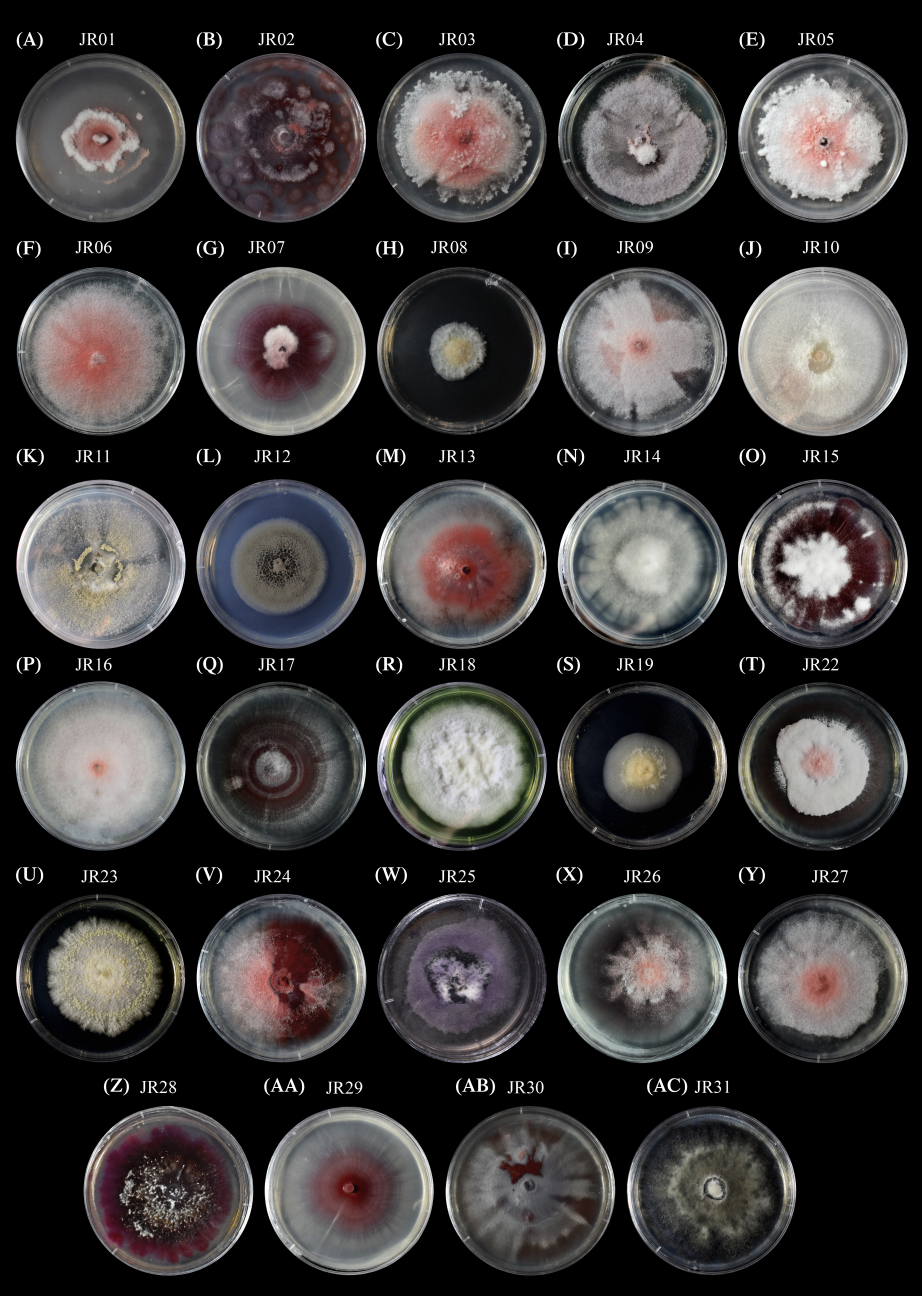
Macroscopic characteristics of fungal isolates growing on Potato‐Dextrose Agar at 50%.

A total of 29 fungal isolates were obtained from *Vanilla* plants, which were characterized based on their macroscopic (colony colour and growth) and microscopic (conidia, appressoria and chlamydospora) characteristics (Table 2). The isolates were grouped into three taxonomic genera, *Fusarium* (25 isolates = 86.2%), *Colletotrichum* (2 isolates = 6.89%) and *Clonostachys* (2 isolates = 6.89%). The isolates differed in their colony characteristics in PDA at 50% (Figure 2). No differences in microscopic structures were observed between isolates molecularly identified as the same species.

**TABLE 2:** Macroscopic description of 29 fungal isolates from commercial crop origin plants (CCO) and crop wild relatives (CWR).

Species	Code	Host	Tissue	Symptoms	Location	Colony characteristics
Fov	JR01	*Vanilla planifolia*	Leaf	x	Buenaventura	Pink hue, cottony mycelium in the centre and creeping towards the margin
*Fusarium*	JR02	*V. planifolia*	Leaf		Buenaventura	Multiple red colonies with white areas, creeping mycelium
*Fusarium concentricum*	JR03	*V. planifolia*	Leaf		Buenaventura	Pink hue with white margin, cottony mycelium
*F. concentricum*	JR04	*Vanilla odorata*	Leaf	x	Buenaventura	Purple‐violet hue, cottony mycelium
*F. concentricum*	JR05	*V. planifolia*	Leaf	x	Buenaventura	White hue with pink centre, cottony mycelium
Fov	JR06	*V. planifolia*	Leaf	x	Buenaventura	Pink hue with white margin, powdery mycelium
Fov	JR07	*V. planifolia*	Leaf	x	Alcalá	White tone with purple‐violet centre and concentric rings, creeping mycelium
*Fusarium solani*	JR08	*V. planifolia*	Leaf	x	Buenaventura	White tone with yellow centre, creeping mycelium, with elevations and irregular margin
*F. solani*	JR09	*V. planifolia*	Leaf		Buenaventura	White tone with pink areas, powdery mycelium
*F. solani*	JR10	*V. planifolia*	Leaf	x	Buenaventura	White tone with pale yellow areas, creeping mycelium
*Fusarium oxysporum* f. sp. *radices‐lycopersici*	JR13	*V. planifolia*	Leaf		Buenaventura	Pink‐red hue with white margin, creeping mycelium
*F. solani*	JR14	*V. planifolia*	Leaf	x	Buenaventura	White‐greyish hue, cottony and striated mycelium
Fov	JR15	*V. planifolia*	Leaf	x	Alcalá	Burgundy tone, with white areas, creeping mycelium with cottony areas
Fov	JR16	*V. planifolia*	Root	x	Alcalá	Pale pink hue, powdery mycelium
*F. oxysporum* f. sp. *radicis‐lycopersici*	JR17	*V. planifolia*	Leaf	x	Buenaventura	Burgundy tone, with white areas and concentric rings, creeping mycelium
*F. solani*	JR19	*Vanilla calyculata*	Leaf	x	Atuncela‐Dagua	White tone with yellow centre, creeping mycelium, with elevations and entire margin
*Fusarium pseudocircinatum*	JR22	*V. planifolia*	Leaf	x	Buenaventura	Pale pink hue, dense cottony mycelium with creeping margin
*F. pseudocircinatum*	JR23	*V. planifolia*	Leaf		Buenaventura	White tone with yellow areas, cottony mycelium, with the presence of exudates
*F. pseudocircinatum*	JR24	*V. planifolia*	Leaf		Buenaventura	Pink‐red hue, creeping mycelium with cottony areas
*F. pseudocircinatum*	JR25	*V. planifolia*	Leaf	x	Buenaventura	Purple hue, cottony mycelium
Fov	JR26	*V. planifolia*	Root	x	Alcalá	Pinkish‐violet hue, cottony mycelium in the centre and creeping towards the margin
Fov	JR28	*V. planifolia*	Leaf	x	Alcalá	Bright purple‐violet hue with white areas, creeping mycelium
*F. oxysporum* f. sp. *radices‐lycopersici*	JR29	*V. planifolia*	Leaf	x	Alcalá	White tone with red centre, creeping mycelium
Fov	JR30	*V. planifolia*	Root	x	Alcalá	Greyish hue with red areas and concentric rings, creeping mycelium
*F. solani*	JR31	*V. planifolia*	Leaf		Buenaventura	Black tone with grey and green areas, cottony mycelium
*Colletotrichum graminicola*	JR12	*V. planifolia*	Leaf		Buenaventura	Opaque green hue, powdery mycelium with presence of invaginations
*Colletotrichum brevisporum*	JR27	*V. odorata*	Leaf		Buenaventura	White tone with pink centre, cottony mycelium
*Clonostachys rosea*	JR11	*V. odorata*	Leaf		Alcalá	Greyish hue with yellow areas, cottony mycelium
*C. rosea*	JR18	*V. planifolia*	Leaf		Alcalá	White tone with yellow pigmentation in the culture medium, dense cottony mycelium

*Note*: Alcalá: CCO; Buenaventura and Atuncela‐Dagua: CWR.

### Fusarium *isolates*

**FIGURE 3:**
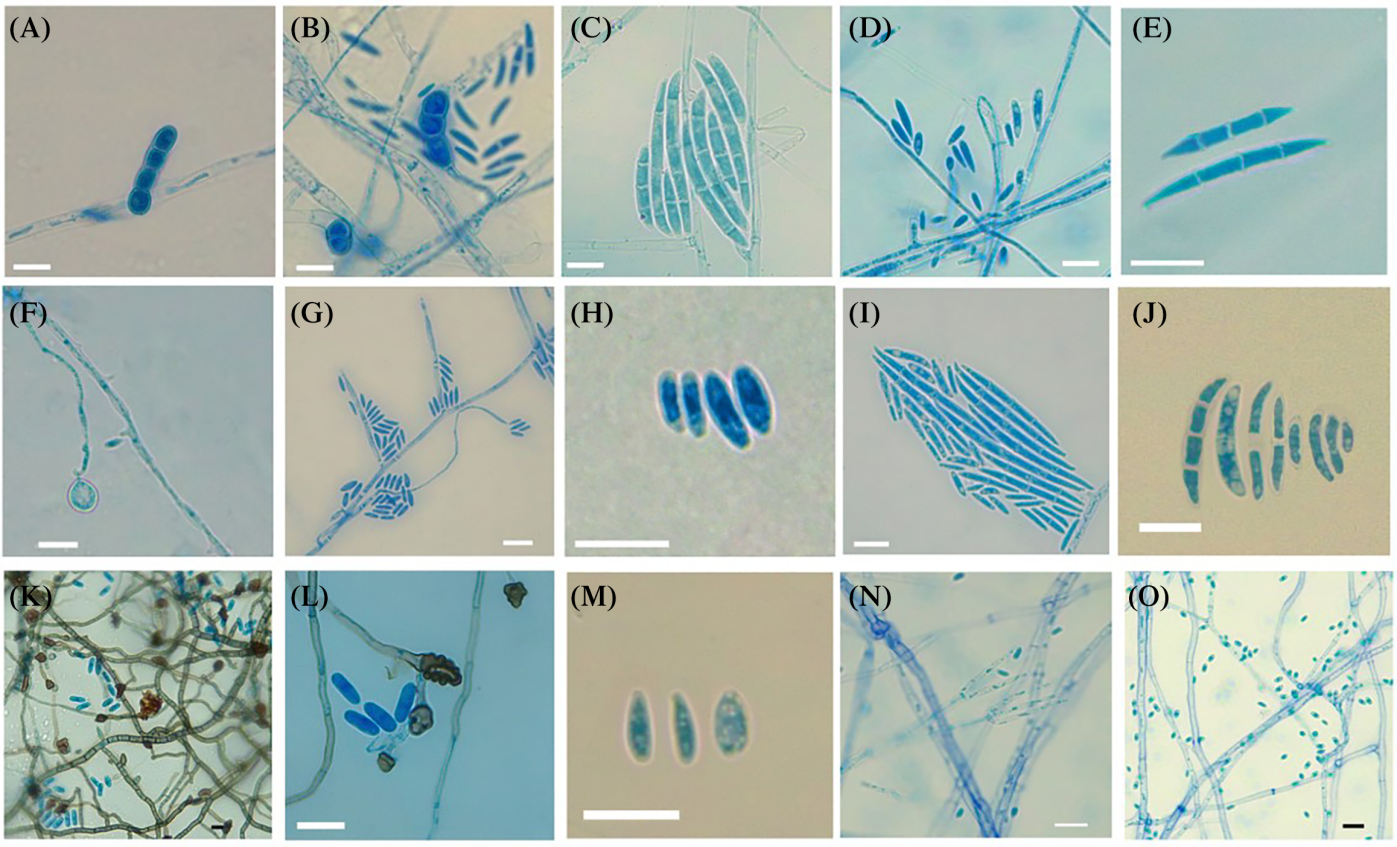
Microscopic characteristics of fungal isolates. (A, B) *Fusarium oxysporum* conidia. (C, D) *Fusarium solani* chlamydospores. (E) *F*. *solani* conidia. (F) *Fusarium pseudocircinatum* chlamydospora. (G, H) *F*. *pseudocircinatum* conidia. (I, J) Conidia of *Fusarium concentricum*. (K, L) Conidia and appressoria *Colletotrichum graminicola*. (M) Conidia *Colletotrichum brevisporum*. (N) Phialides *Clonostachys rosea*. (O) Conidia *C. rosea* (scale bar = 10 μm).

For *F. oxysporum* isolates, characteristics observed included single‐celled microconidia of variable shape (cylindrical, ovoid or reniform), straight to slightly curved, size 1.1–10.0 × 1.0–3.3 μm, fusiform bicellular mesoconidia, without curvature, size 4. 3–19.3 × 3.0–2.7 μm and falcate macroconidia with 3–4 septa, size 14.7–46.2 × 1.3–5.1 μm, which presented blunt cells at both ends, these isolates did not generate chlamydospores (Figure 3A,B). *F. solani* isolates showed a slight difference to *F. oxysporum* with respect to mesoconidia and microconidia. However, *F*. *solani* isolates generated abundant intercalary and terminal chlamydospores in chains. In addition, macroconidia with size of 10.4–48.1 × 2.9–5.6 μm were observed slightly wider and presented 2–3 septa (Figure 3C–E).

In *F. pseudocircinatum* isolates, unicellular microconidia were observed with a cylindrical shape, size of 2.5–10.1 × 1.0–2.6 μm, as well as terminal solitary chlamydospores (Figure 3F–H). For *Fusarium concentricum* isolates, spindle‐shaped unicellular microconidia, size 2.7–10.7 × 1.1–2.6 μm and macroconidia, with 2–3 septa, size 10.5–27.3 × 1.0–2.9 μm, which presented papillary cells at both ends, no mesoconidia or chlamydospores were observed for these isolates (Figure 3I,J).

### *Other isolates obtained from* Vanilla *spp.*

For the isolate of *Colletotrichum graminicola*, smooth hyaline conidia were observed, with oval shape, rounded edges at both ends and size of 7.2–15.8 × 2.2–6.2 μm, dematiaceous appressoria were observed, solitary or grouped, with oval or fusiform shape, size of 1.2–4.1 × 2.9–9.6 μm (Figure 3K,L). For the isolate of *Colletotrichum brevisporum*, single‐celled hyaline conidia of variable shape (cylindrical, ovoid, fusiform) were observed, presenting one rounded and one acute end; sometimes with visible vacuoles in the cytoplasm, size 2.9–6.9 × 1.0–2.7 μm; the isolation of *C. brevisporum* did not generate appressoria (Figure 3M). For *Clonostachys rosea* isolates, branched conidiophores with 2–4 phialides, hyaline conidia, size 1.7–6.9 × 1.6–4.0 μm, scattered or clustered, oval to elliptical in shape were observed (Figure 3N,O).

### Molecular and phylogenetic characterization of fungal isolates

DNA sequences were obtained from the ITS‐rRNA (~550 bp) and EF1‐α (~700 bp) loci from 27 of the 29 fungal isolates (Table 3); isolate JR02 did not amplify for either locus and was identified only based on morphological characteristics (Table 2). JR12 did not amplify for the ITS‐rRNA locus, and JR09 did not amplify for the EF1‐α locus. Of the isolates sequenced, 24 correspond by molecular identification to the genus *Fusarium*: *F. oxysporum*, with 11 isolates (37.9%); *F. solani*, with 6 isolates (20.7%); *F*. *pseudocircinatum*, with 4 isolates (13.8%); and *F. concentricum*, with 3 isolates (10.3%). The remaining isolates corresponded to *C. rosea*, with two isolates (6.89%); *C. brevisporum*, with one isolate (3.44%); and *C. graminicola*, with one isolate (3.44%). All sequences analysed showed a similarity equal or major to 97% with respect to the type sequences deposited in GenBank, according to their availability.

**TABLE 3:** Results of BLAST analysis for sequences of the ITS‐rRNA and EF1‐α loci.

Isolate code	ITS				EF1‐α			
	Identified species	Accession deposited in GenBank	% similitude	Closest GB match	Identified species	Accession deposited in GenBank	% similitude	Closest GB match
JR01	Fov	PP033028	100	MT560381	Fov	PP835904		
JR02	*Fusarium*	No data	No data	No data	*Fusarium*	No data	No data	No data
JR03	*Fusarium concentricum*	PP033029	100	MH862659	*F. concentricum*	PP835905	99.8	AF160282
JR04	*F. concentricum*	PP033030	100	NR_111886	*F. concentricum*	PP835906	99.7	MT010992
JR05	*F. concentricum*	PP033031	100	MH862659	*F. concentricum*	PP842020	99.37	AF160282
JR06	Fov	PP033032	100	KM268692	Fov	PP754860	99.85	KM065867
JR07	Fov	PP033033	100	KM268692	Fov	PP835908	99.85	KM065856
JR08	*Fusarium solani*	PP033034	97.54	NR_163531	*F. solani*	PP778402	97	HQ731056
JR09	*F. solani*	PP033035	98.92	KT313633	No data	No data	No data	No data
JR10	*F. solani*	PP033036	97	NR_163531	*F. solani*	PP778403	97.63	HE647955
JR11	*Clonostachys rosea*	PP033037	99.82	NR_165993	*Clonostachys* sp.	PP835900		
JR12	No data	No data	No data	No data	*Colletotrichum graminicola*	PP835901	99.07	CP117787
JR13	*Fusarium oxysporum*	PP033038	100	LC460263	*F. oxysporum* f. sp. *radicis‐lycopersici*	PP835909	99.71	HM057327
JR14	*F. solani*	PP033039	97.35	KT313633	*F. solani*	PP778404	97	KT313611
JR15	Fov	PP033040	99.82	MT560381	Fov	PP835910	97.52	KU378600
JR16	Fov	PP033041	100	MW497617	Fov	PP754861	99.71	KM065856
JR17	*F. oxysporum*	PP033042	100	MT560381	*F. oxysporum* f. sp. *radicis‐lycopersici*	PP835911	99.85	HM057296
JR18	*C. rosea*	PP033043	99.8	OQ910800	*Clonostachys* sp.	PP835902	98.75	KY679530
JR19	*F. solani*	PP033044	99.55	NR_163531	*F. solani*	PP778405	97.06	KT313611
JR22	*Fusarium pseudocircinatum*	PP033045	99.8	MG838044	*F. pseudocircinatum*	PP778407	99.12	MG838031
JR23	*F. pseudocircinatum*	PP033046	99.79	MG838044	*F. pseudocircinatum*	PP778408	97	MT011003
JR24	*F. pseudocircinatum*	PP033047	99.8	NR_163683	*F. pseudocircinatum*	PP778409	99.26	KX870039
JR25	*F. pseudocircinatum*	PP033048	100	MG838044	*F. pseudocircinatum*	PP778410	99.27	MT011003
JR26	Fov	PP033049	100	OK509834	Fov	PP754862	99.38	KU378600
JR27	*Colletotrichum brevisporum*	PP033050	98.11	KC790943	*Colletotrichum* sp.	PP835903	100	GU994269
JR28	Fov	PP033051	100	MF993439	Forv	PP754863	99, 71	KM065862
JR29	*F. oxysporum*	PP033052	99.07	MT560381	*F. oxysporum* f. sp. *radicis‐lycopersici*	PP835912	99.85	HM057296
JR30	Fov	PP033053	100	MW798779	Fov	PP754864	97.2	KM065862
JR31	*F. solani*	PP033054	97.06	NR_163531	*F. solani*	PP778406	97	KT313611

**FIGURE 4:**
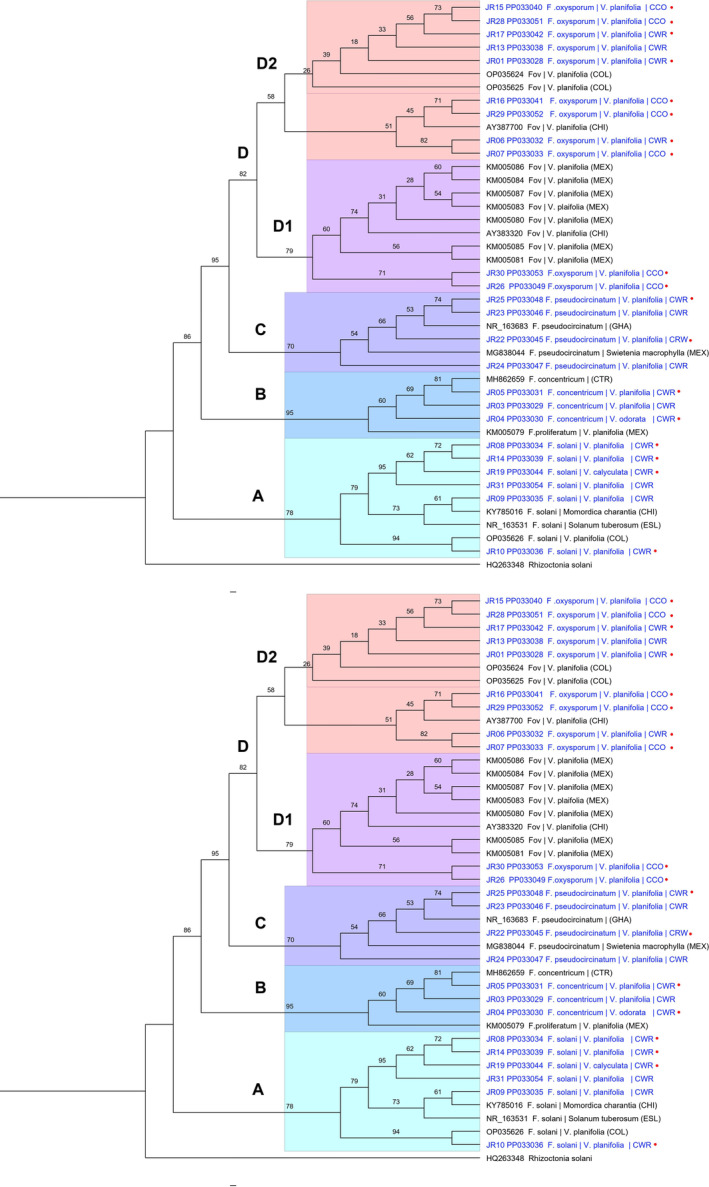
Phylogenetic tree based on the ITS‐rRNA locus of 24 isolates from this study (in blue) and 21 reference sequences from GenBank (Table 1). The tree was inferred from 10,000 replicates, using the maximum likelihood (MV) method based on the TIM2e + G4 model. *Rhizoctonia solani* was chosen as outgroup. The origin of the isolates is indicated: CHI, China; COL, Colombia; CTR, Costa Rica; ESL, Slovenia; GHA, Ghana; MEX, Mexico; REU, Reunion Island. In addition, it is indicated whether the isolates are from commercial crop origin plants or from crop wild relatives. symptomatic tissue.
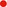

In the phylogenetic analysis for ITS‐rRNA, *Fusarium* isolates were grouped into four clades with a bootstrap support above 70 (Figure 4). Clades A to C contained 12 isolates from vanilla crop wild relatives with molecular affinity to *F. solani* (Clade A), *F. concentricum* and *F. proliferatum* (Clade B), and *F. pseudocircinatum* (Clade C). These isolates were isolated from both asymptomatic and symptomatic tissue.

Clade D grouped 11 isolates together with reference sequences for *F. oxysporum*, including isolates of Fov from studies of vanilla in Mexico, China and Colombia. These included JR16, JR26 and JR30, isolated from symptomatic root tissue sampled from CCO plants. Other isolates in this clade came from leaf tissue sampled from CCO material (JR07, JR15, JR28, JR29) and from crop wild relatives (JR01, JR06, JR13, JR17). All isolates in this clade were derived from symptomatic tissue. In Clade D, two subclades can be distinguished, indicating two distinct genotypes, although one with a lower bootstrap value. Both subclades contained isolates from vanilla in Colombia and China, while isolates from Mexico were limited to subclade D2.

**FIGURE 5:**
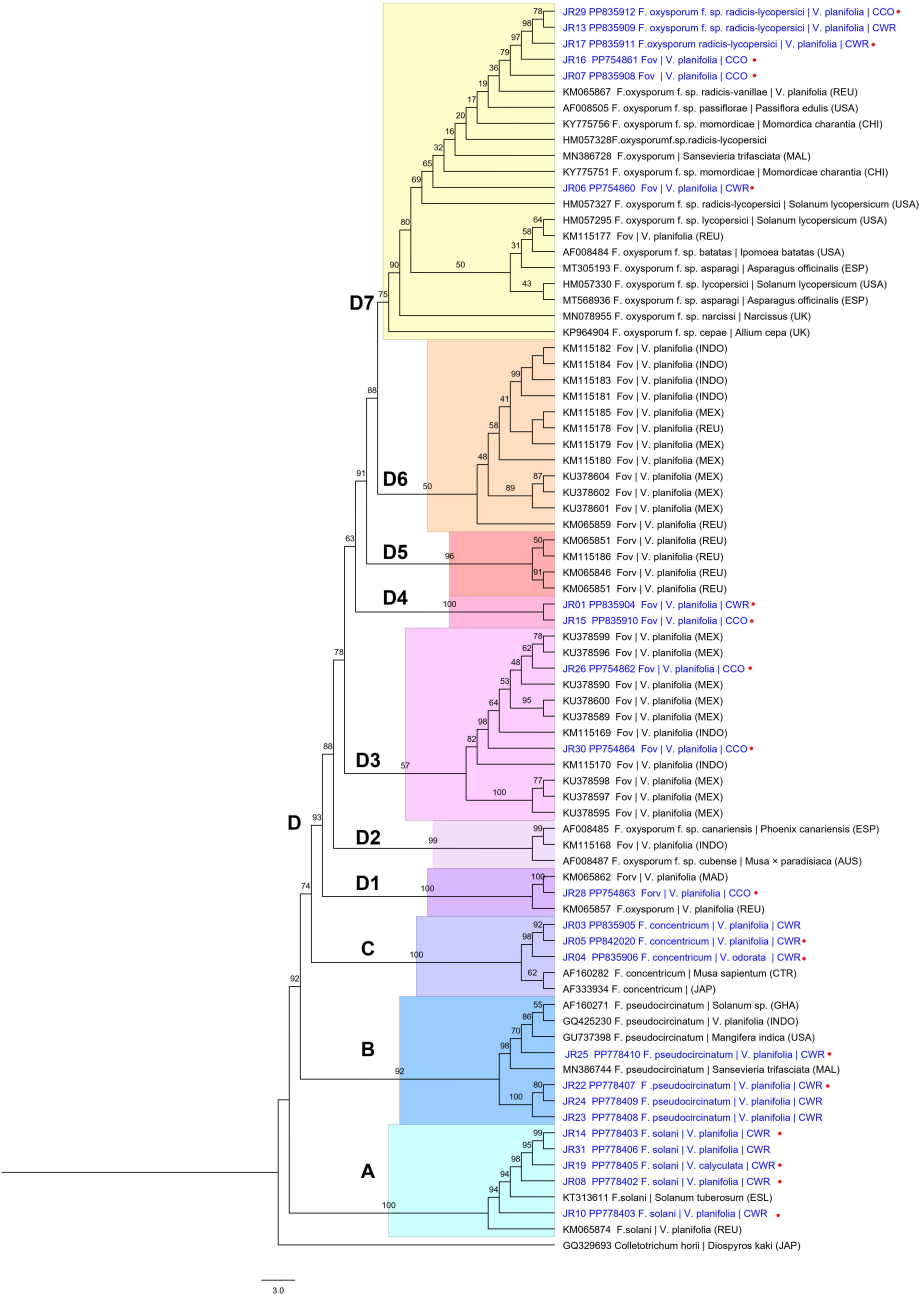
Phylogenetic tree based on the EF1‐α locus of 23 isolates from this study (in blue) and 54 GenBank reference sequences with the accession code (Table 1). The tree was inferred from 10,000 replicates using the maximum likelihood (MV) method based on the HKY + G4 model chosen according to BIC. One isolate of *Colletotrichum horii* was chosen as outgroup. The origin of the isolates is indicated: AUS, Australia; CHI, China; CTR, Costa Rica; ESL, Slovenia; ESP, Spain; INDO, Indonesia; JAP, JAPAN; MAD, Madagascar; MAL, Malaysia; MEX, Mexico; REU, Reunion Island; UK, United Kingdom; USA, United States. Additionally, it is noted whether the isolates are from commercial crop origin plants or from crop wild relatives. symptomatic tissue.
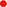

The phylogenetic analysis with the EF1‐α sequence data also yielded four main clades, with bootstrap supports greater than 90 (Figure 5). Similarly to the ITS‐rRNA data, three clades corresponded to isolates from vanilla crop wild relatives with molecular affinity to *F. solani* (Clade A), *F. pseudocircinatum* (Clade B) and *F. concentricum* (Clade C).

Clade D grouped the 11 isolates of *F. oxysporum* from this study along with isolates from vanilla and other crops from various regions around the world. Greater resolution was observed with this locus, with seven distinguishable subclades (5 with bootstrap values greater than 50).

Subclade D1 included an isolate from CCO plants leaf tissue (JR28) that clustered with a genotype of *F. oxysporum* f. sp. *radicis‐vanillae* (Forv) from Madagascar (KM065862) and another isolate from Reunion (KM065857). Subclade D2 also includes three isolates corresponding to *F. oxysporum* f. sp. *canariensis* (AF008485), (KM115168) and *F. oxysporum* f. sp. *cubense* (AF008487). Subclade D3 grouped the isolates JR26 and JR30 from symptomatic root tissue along with 8 isolates of *Fov* from Mexico (KU378589, KU378590, KU378595‐KU378600) and two from Indonesia (KM115169, KM115170). Subclade D4 is composed exclusively of isolates JR01 and JR15 from *V. planifolia*. In Subclade D5, three isolates of *Forv* (KM065846, KM065851, KM065853) and one isolate of *Fov* (KM115186) from Reunion are present. Subclade D6 included 12 isolates of *Fov* from Indonesia (KM115181‐KM115184), Mexico (KM115179, KM115180, KM115185, KU378601, KU378602, KU378604) and Reunion (KM065859, KM115178). Subclade D7 groups six isolates from the present study, derived from both asymptomatic and symptomatic tissue from CCO plant material and CWR. Three of these isolates are identified as Fov (JR06, JR07, JR16), and the other three as *F. oxysporum* f. sp. *radicis‐lycopersici* (JR13, JR17, JR29), along with isolates of various FOSC special forms distributed across different crops worldwide.

Samples from vanilla studies in different geographic regions (Mexico, Indonesia and Reunion) were also dispersed across different subclades, evidencing multiple genotypes in each region. The isolates of *F. oxysporum* f. sp. *vanillae* (Fov) from Indonesia, Mexico and Reunion were dispersed in subclades D2, D3, D5, D6 and D7, while the isolates of *F. oxysporum* f. sp. *radicis‐vanillae* (Forv) from Reunion were also present in D1, D5 and D6.

The isolates from the present study were distributed across four of these subclades: D1, D3, D4 and D7. In this way, the isolates from the present study cluster with genotypes of fov in D3 and D7. One isolate, JR28, from leaf material in CCO plants, clustered with the forv genotype from Madagascar in D.

## DISCUSSION

In this study, we isolated fungi from both symptomatic and asymptomatic tissue from plants in a vanilla crop and crop wild relatives (CWR) in Colombia. Molecular identification with ITS‐rRNA and EF1‐α loci together with morphological characterization showed the presence of three fungal genera, *Colletotrichum* (6.89%), *Clonostachys* (6.89%) and *Fusarium* (86.2%), the latter being the most relevant in the development of phytosanitary problems in the vanilla crop (Adame‐García et al., 2015; Hernández‐Hernández, 2019).

This is the first study to show the presence of a fungal isolate from vanilla with affinity to *F. oxysporum* f. sp. *radicis‐vanillae* (Forv). The fungal strain JR28, isolated from necrotic tissue of a leaf from a vanilla plant, showed affinity in the EF1‐α locus to a Forv isolate from Madagascar (Koyyappurath et al., 2016). Interestingly, in the ITS locus, the sequence of this isolate grouped with several other isolates from Colombia and an isolate identified as *F. oxysporum* f. sp. *vanillae* (Fov) from China.

Our findings complement the report of Fov in Colombia (Mosquera‐Espinosa et al., 2022), where *F. oxysporum* is associated with disease symptoms in vanilla. This new study aimed to define whether genetic variants of the pathogen species can be identified using molecular markers. This led to the discovery of the presence of fungi with affinity for Fov and Forv, in both the roots of vanilla CCO plants with root and stem wilt, and in leaf material from CCO plants and CWR, whether symptomatic or asymptomatic.

Pathogenicity studies previously conducted to evaluate Fov isolates have shown that this special form is highly pathogenic on *V. planifolia* (Adame‐García et al., 2015; Koyyappurath et al., 2015, 2016; Pinaria et al., 2015). However, studies conducted by Mosquera‐Espinosa et al. (2022) and Manrique‐Barros et al. (2023) demonstrate that pathogenicity test results can vary due to environmental factors, even when performed under in vitro conditions. Additionally, the loss of genetic variability resulting in reduced expression of resistance genes in plants, due to continuous micropropagation processes, can lead to erroneous diagnoses (Porras‐Alfaro & Bayman, 2011; Zhang et al., 2019). These findings raise concerns about accurate diagnosis in the emerging vanilla crop in Colombia, highlighting the need to continue molecular diagnostic testing to ensure timely monitoring and to propose integrated management strategies for Fusarium wilt.

This research represents the initial effort to jointly generate sequences of the ITS‐rRNA and EF1‐α regions for *Fusarium* isolates from *V. planifolia* and its CWR in Colombia. At both loci, the isolates obtained of *F. oxysporum* were grouped within a clade together with accessions of both Fov and Forv obtained from *V. planifolia*. Besides, the coexistence of different *Fusarium* species was registered in vanilla crop wild relatives. Therefore, the first hypothesis raised in this study is accepted, since the presence of Fov within the microbial community associated with vanilla in agroforestry systems is confirmed.

Fungal isolates were obtained with affinity to other *Fusarium* species: *F. solani*, *F. concentricum* and *F. pseudocircinatum*, which are reported to cause different types of rots in a wide variety of commercial crops (Aoki et al., 2014; Huang, 2009; Koyyappurath et al., 2015; O'Donnell et al., 2004; Santillán‐Mendoza et al., 2018). These species have been previously reported in vanilla‐producing countries in Central America, Africa and Asia, which evidences the wide geographic distribution of the vanilla microbiota (Adame‐García et al., 2015; Agrios, 2005; Koyyappurath et al., 2016; Pinaria et al., 2015).

*F. solani* has been previously reported in commercial vanilla crops in Indonesia (Pinaria et al., 2015), Reunion (Koyyappurath et al., 2016) and Colombia (Mosquera‐Espinosa et al., 2022). Based on the prevalence and pathogenicity studies in vanilla, *F. solani* has been previously considered as a secondary pathogen that induces mild symptoms on *Vanilla* spp. (Mosquera‐Espinosa et al., 2022; Pinaria et al., 2015).

Within the *F. fujikuroi* species complex (FFSC), the species *F. concentricum* is part of the Asian clade, while *F*. *pseudocircinatum* is in the African clade (O'Donnell, Cigelnik, et al., 1998; Yilmaz et al., 2021). Fungi that are part of this species complex are reported to cause important diseases in commercial crops such as ‘bakanae’ or ‘dumb seedling disease’ in rice (*Oryza sativa*) (Drenkhan et al., 2020), pitch canker in pine (*Pinus* spp.) (Kang et al., 2019) and stalk rot in maize (*Zea mays*) (Chang et al., 2020). Interestingly, in this study, these three species were not isolated from the vanilla crop, being derived from both symptomatic and asymptomatic tissue in crop wild relatives. Given their low frequency of occurrence and reduced ability to induce symptoms in vanilla in Reunion Island (Koyyappurath et al., 2015) and Indonesia (Koyyappurath et al., 2016; Pinaria et al., 2010), it has been proposed that their main role maybe as saprophytes or endophytes in this host (Koyyappurath et al., 2016). Nonetheless, more work is needed to determine whether, and under what conditions these *Fusarium* species may develop pathogenic traits in vanilla.

In the present study, the macroscopic characteristics of the colonies, as well as the results obtained from the analysis of the EF1‐α region, indicate a high degree of variation among Fov isolates from the primary and secondary gene pool of *V*. *planifolia*, in agreement with other studies that report the increase in the number of lineages of the pathogen in natural host distribution centres (Banke et al., 2004; Grünwald & Flier, 2005; Stukenbrock et al., 2007), as is the case of *F. oxysporum* f. sp. *cubense* in Indonesian *Musa* varieties (Maryani et al., 2018; O'Donnell, Kistler, et al., 1998). It should be noted that Colombia is in the centre of neotropical diversity of the genus *Vanilla*. Therefore, we suggest the need to evaluate a hypothesis regarding potential coevolution between the causal agent of Fusarium wilt and close relatives of the vanilla crop.

The EF‐1α locus revealed greater genetic variation and a higher degree of resolution of different genotypes, proving useful for resolving interspecific phylogenetic relationships within FOSC (Lombard, Sandoval‐Denis, Cai, et al., 2019). Genetic differentiation was observed between the Forv and Fov forms of *F. oxysporum*. These results allow for the acceptance of the second proposed hypothesis, as the ITS‐rRNA and EF‐1α loci provided sufficient phylogenetic resolution to delimit the special forms Fov and Forv. However, caution may be necessary when using EF‐1α for molecular diagnosis of special forms (Thangavel et al., 2021), as clonal lineages of *F. oxysporum* may have incongruent conserved genes (Maryani et al., 2018; Mat Razali et al., 2019).

On the contrary, plant pathogenic strains of the *F. oxysporum* species present genes encoding for effector proteins secreted into the host xylem (‘secreted into the xylem’: SIX), which are unique among special forms given that they contribute to pathogenicity and virulence (Batson et al., 2021; Jangir et al., 2021; Thangavel et al., 2021). In a study conducted on passion fruit (*Passiflora edulis*) plants, Thangavel et al. (2021) analysed the phylogenetic relationships of *F. oxysporum* f. sp. *passiflora* (FOP) using EF‐1α, β‐tubulin and the effector genes *SIX6* and *SIX9*. It was determined that the EF‐1α and β‐tubulin loci are not sufficient to resolve in certain cases the identity of FOP, while the SIX genes generate stronger clades with greater support in phylogenetic analyses. Other studies propose that SIX genes (*SIX1*, *SIX5*, *SIX6*, *SIX7*, *SIX8*, *SIX9*, *SIX10*, *SIX11* and *SIX14*) are crucial for discriminating the potential ecological roles of *F. oxysporum* strains (Batson et al., 2021; Jangir et al., 2021), as non‐pathogenic strains from soil in natural ecosystems partially or completely lack SIX effector genes (Jangir et al., 2021; Rocha et al., 2016). It is necessary to clarify that, for the specific case of Fov and Forv, as well as in other special forms, SIX gene sequences have not yet been identified (Jangir et al., 2021). Therefore, further research is required to obtain sequences of these genes and thus deepen the understanding of the interaction between the special forms of *F. oxysporum* and vanilla plants.

Regarding the microscopic characteristics of *Fusarium* isolates, differences in the shape and size of conidia, the number of septa and the formation of chlamydospores were evidenced among species. Despite this, the ambiguity of morphological characters for identification down to species remains an obstacle for early plant diagnosis based on this trait alone, even more so considering that there are areas with high diversity of the genus *Fusarium* (Crous et al., 2015; T. R. Gordon, 2017; Lombard, Sandoval‐Denis, Cai, et al., 2019; Lombard, Sandoval‐Denis, Lamprecht, et al., 2019). Therefore, thorough and exhaustive morphological examinations should be complemented with the use of specific molecular markers and multigene phylogenetic analyses, including SIX genes, EF‐1α and ITS‐rRNA (Carbajal‐Valenzuela et al., 2022; Jangir et al., 2021; O'Donnell, Kistler, et al., 1998). The latter locus currently has widespread application as a universal barcode marker for fungal identification (Schoch et al., 2012) and is used in metabarcoding studies, including those on vanilla (Carbajal‐Valenzuela et al., 2022).

In conclusion, through molecular identification using both the EF‐1α and ITS‐rRNA loci of fungal isolates from symptomatic and asymptomatic tissues of commercial vanilla plants and crop wild relatives (CWR), we documented the presence of known vanilla pathogens, specifically *F. oxysporum* f. sp. *vanillae* (Fov) and *F. oxysporum* f. sp. *radicis‐vanillae* (Forv), in Colombia. The EF‐1α locus provided greater genotype resolution, with reference sequences available for Forv. However, the ITS‐rRNA locus is widely applied. We recommend the continued use of both loci for Fusarium diagnosis in vanilla, including their application to emerging vanilla crops in countries such as Colombia. Early detection of potential fungal pathogens will facilitate the development of effective integrated crop management strategies.

## AUTHOR CONTRIBUTIONS

**Jayerlin Rodríguez‐Bastidas:** Data curation; formal analysis; writing – original draft; methodology; investigation; writing – review and editing; visualization; software. **Santiago Manrique‐Barros:** Methodology; writing – review and editing; visualization. **Donald Riascos‐Ortiz:** Formal analysis; writing – review and editing; software. **Ana T. Mosquera‐Espinosa:** Conceptualization; writing – original draft; supervision; project administration; writing – review and editing. **Nicola S. Flanagan:** Conceptualization; formal analysis; writing – original draft; investigation; supervision; project administration; writing – review and editing; validation; funding acquisition; resources.

## CONFLICT OF INTEREST STATEMENT

The authors declare no conflicts of interest.

## Supporting information


**Data S1.** Supporting information.

## Data Availability

Sequence data generated in this study have been submitted to the National Center for Biotechnology Information under accession numbers PP033028‐PP033054, PP754860‐PP754864, PP778402‐PP778410 y PP835900‐PP835912. Other data presented in this study can be requested from the corresponding author.
